# Severity of SARS-CoV-2 Omicron XBB subvariants in Singapore

**DOI:** 10.1016/j.lanwpc.2023.100849

**Published:** 2023-07-25

**Authors:** Rachael Pung, Xin Peng Kong, Lin Cui, Sae-Rom Chae, Mark I-Cheng Chen, Vernon J. Lee, Zheng Jie Marc Ho

**Affiliations:** a Ministry of Health, Singapore; b Centre for the Mathematical Modelling of Infectious Diseases, London School of Hygiene and Tropical Medicine, United Kingdom; c National Centre for Infectious Diseases, Singapore; d Saw Swee Hock School of Public Health, National University of Singapore, Singapore

## Abstract

Several XBB subvariants such as XBB.1.5, XBB.1.9, XBB.1.16 and XBB.2.3 co-circulate in Singapore. Despite the different viral properties of XBB.1.16 as compared to other XBB subvariants, comparison on their severity is limited. In this study, we investigate the outcomes of hospitalisation and severe COVID-19 infection in individuals infected with different XBB subvariants, adjusted for potential confounders such as age and vaccination history. Overall, our preliminary analysis showed that the risk of severe outcomes when infected with XBB.1.16 is higher than that of XBB.1.5 or XBB.1.9 but there is no difference in the risk of hospitalisation across different XBB subvariants.

## Main

SARS-CoV-2 Omicron sublineage XBB is a recombinant of BA.2.10.1 and BA.2.75 sublineages and was first identified in August 2022.^1^ This strain led to a major outbreak in Singapore which peaked in October 2022 before falling to a low base in early 2023. Since then, several XBB subvariants such as XBB.1.5, XBB.1.9, XBB.1.16 and XBB.2.3 have emerged. While they have similar genetic profiles, XBB.1.16 has been designated as a variant of interest by WHO on 17 Apr, 2023 given its growth advantage and immune escape properties.^2^^,^^3^ Unlike previous COVID-19 outbreaks where a sustained rise in cases was attributed to one dominant SARS-CoV-2 variant, the increase in COVID-19 cases in Singapore is now predominantly driven by these four XBB subvariants emerging at different time points but presenting as one confluent outbreak that started in early March 2023. Despite the different viral properties of XBB.1.16 as compared to other XBB subvariants, comparison on their severity is limited. As such, it is unclear whether an increase in the number of hospitalised cases is an outcome of faster transmission or higher severity or both. In this study, we investigated the outcomes of hospitalisation and severe COVID-19 infection in individuals infected with different XBB subvariants, adjusted for potential confounders such as age and vaccination history. Findings of this study would be useful for the development of vaccines.

In Singapore, whole genome sequencing (WGS) is performed on a subset of confirmed COVID-19 cases (both locally infected and imported cases) who tested positive via PCR test administered by a healthcare provider. From 1 January to 27 May 2023, WGS was performed on respiratory samples from 7733 cases of which 5910 were infected with an XBB subvariant (Table 1)—1210 (20%) were XBB.1.16, 1088 (18%) were XBB.1.5, 1332 (23%) were XBB.1.9, 1305 (22%) were XBB.2.3, and the remaining 975 (16%) were other XBB sublineages (Table 1).

**Table 1:** Characteristics of XBB cases by vaccination history and clinical outcomes.

Vaccination history	Non-hospitalised	Non-severe hospitalised	Severe hospitalised
XBB.1.16 (n = 1210) *Detected from 15 Feb, 2023*			
Primary series with booster <1 year	374	122	63
Primary series with booster ≥1 year	244	85	49
At most primary series	158	89	26
Total	776 (64%)	296 (25%)	138 (11%)
XBB.1.5 (n = 1088) *Detected from 5 Dec, 2022*			
Primary series with booster <1 year	316	144	49
Primary series with booster ≥1 year	250	94	38
At most primary series	95	84	18
Total	661 (61%)	322 (29%)	105 (10%)
XBB.1.9 (n = 1332) *Detected from 6 Jan, 2023*			
Primary series with booster <1 year	370	171	53
Primary series with booster ≥1 year	305	130	43
At most primary series	116	116	28
Total	791 (60%)	417 (31%)	124 (9%)
XBB.2.3 (n = 1305) *Detected from 20 Feb, 2023*			
Primary series with booster <1 year	395	158	62
Primary series with booster ≥1 year	253	118	58
At most primary series	133	99	29
Total	781 (60%)	375 (29%)	149 (11%)
Other XBB (n = 975) *Detected from 6 Sep, 2023*			
Primary series with booster <1 year	296	135	37
Primary series with booster ≥1 year	187	99	28
At most primary series	113	73	7
Total	596 (61%)	307 (32%)	72 (7%)

Multivariate logistic-regression was used to investigate the (i) severity and (ii) outcomes of hospitalisation due to infection with different SARS-CoV-2 Omicron XBB subvariants, after adjusting for age and vaccination history. A severe infection is defined as a hospitalised case who either required supplemental oxygen, was admitted to ICU or died, while a non-severe infection is defined as all other hospitalised or non-hospitalised cases. The vaccination history of a case is classified as (i) completed the primary vaccination series and had a booster in less than 1 year prior to the current infection episode, (ii) completed the primary vaccination series and had a booster 1 year or more prior to the current infection episode, (iii) at most completed the primary vaccination series.

**Fig. 1:**
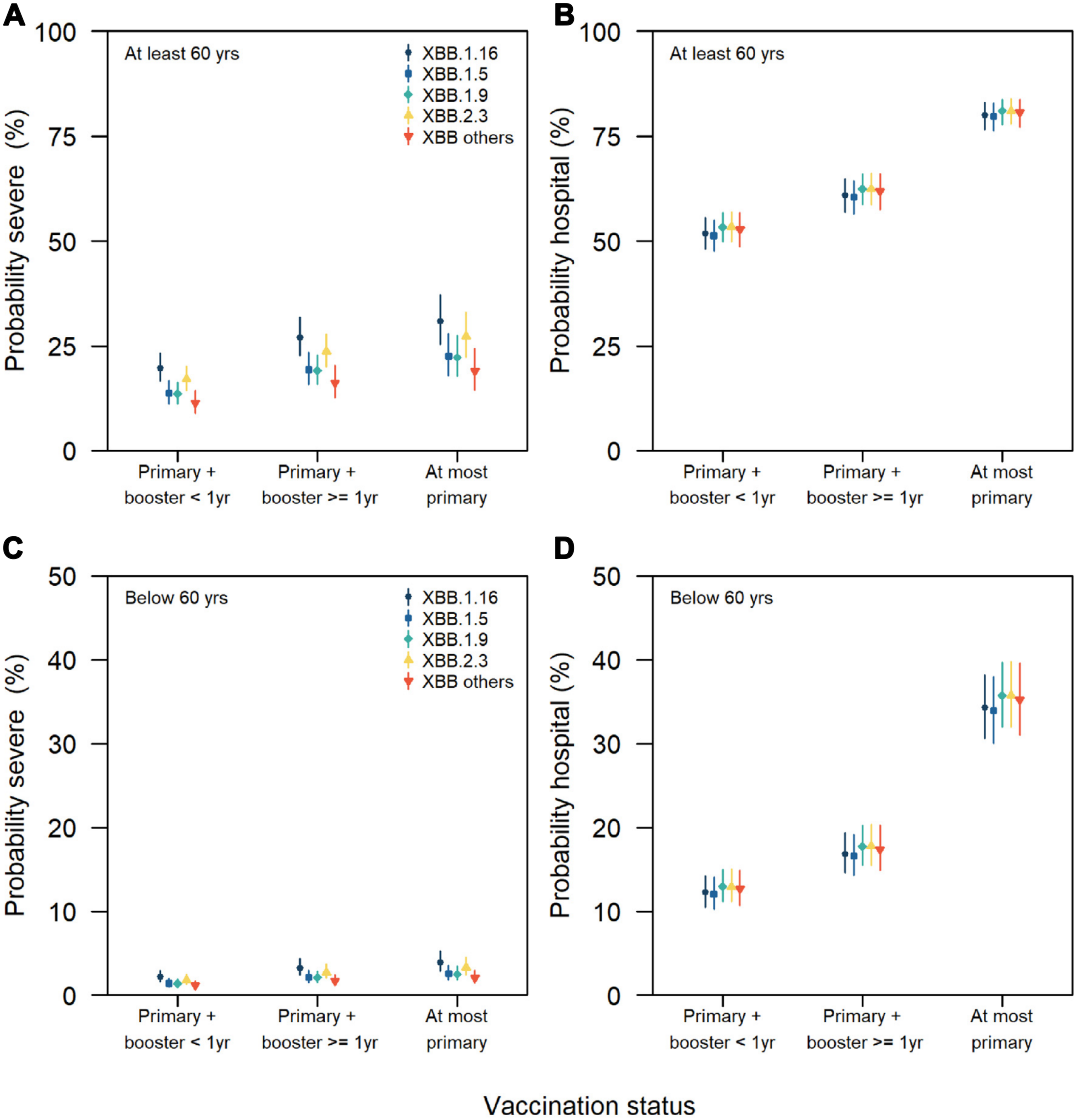
Probability of infection outcomes for different XBB subvariants and vaccination history; (A) severe infection in those aged 60 and above, (B) hospitalisation in those aged 60 and above, (C) severe infection in those aged below 60, (D) hospitalisation in those aged below 60.

After adjusting for the confounders, we estimated a higher risk of severe outcomes when infected with XBB.1.16 as compared to XBB.1.5 or XBB.1.9 but no difference when compared to XBB.2.3 (Fig. 1A and C). Furthermore, we observed no significant differences in the risk of COVID-19 hospitalisation outcomes across different XBB subvariants (Fig. 1B and D). Regardless of the XBB subvariant of infection, the risk of hospitalisation was about 1.5 times higher in those who at most completed their primary vaccination as compared to those with primary vaccination and a booster (Fig. 1B and D). Given that vaccination history of imported cases may be incomplete, sensitivity analysis was performed using local cases only and similar findings were observed.

Our analysis has some limitations. Given the low number of hospitalised COVID-19 cases, a higher proportion (60–90%) of these cases were sampled for WGS. In contrast, about 10% of the non-hospitalised cases were sampled for WGS. However, this sampling criteria is consistent across the period of study. Accounting for this sampling bias would increase the proportion of non-hospitalised cases in Table 1, but would not affect the mean outcomes of the multivariate logistic regression. Delays in the onset of severe infection may bias the severity of XBB.1.16 and XBB.2.3 downwards, given that these two variants were only detected in samples from mid February 2023 onwards. In the current dataset, about 60–70% of the hospitalised XBB.1.16 and XBB.2.3 cases were notified before April, while this was 60% in the non-hospitalised XBB.1.16 and XBB.2.3 cases. Furthermore, the number of COVID-19 hospitalised cases by end May 2023 was a third of the estimated cases at the peak of the outbreak in April 2023.^4^ Thus, the impact of delayed outcome is not likely to have had a large effect on the analysis.

Overall, our preliminary analysis showed a higher risk of severe outcomes when infected with XBB.1.16 as compared to XBB.1.5 or XBB.1.9 but no difference in the risk of hospitalisation across different XBB variants. As countries scale down on COVID-19 testing and reporting, studies to distinguish the growth advantage of different COVID-19 variants would be increasingly challenging given the drop in case ascertainment, lack of contact exposure data and studies on the impact of hybrid immunity on the risk of transmission.^5^ However, continued data collection on the clinical severity of cases and such comparative analyses would help to identify changes in severity of SARS-CoV-2 variants and hence factors contributing to the growth in COVID-19 hospitalisations.

## Contributors

Conceptualization: R.P. and Z.J.H.M. Methodology: R.P. and X.P.K. Investigation: R.P., X.P.K., and L.C. Visualization: R.P. and X.P.K. Supervision: V.J.L. and Z.J.H.M. Writing, original draft: R.P. and X.P.K. Writing, review and editing: all authors. All authors read and approved the final manuscript.

## Ethics

The study was approved by the London School of Hygiene & Tropical Medicine Observational Research Ethics Committee (ref. 25727). All data and analysis were collected and performed in line with the Infectious Diseases Act in Singapore which permits the collection and publication of surveillance data.

## Declaration of interests

The authors declare no competing interest.
